# Laser pulse-electron beam synergy effect on electron self-injection and higher energy gain in laser wakefield accelerators

**DOI:** 10.1038/s41598-020-79556-9

**Published:** 2021-01-08

**Authors:** Sahar Barzegar, Ali Reza Niknam

**Affiliations:** Laser and Plasma Research Institute, Shahid Beheshti University, 1983969411 Tehran, Iran

**Keywords:** Plasma-based accelerators, Plasma physics

## Abstract

A new scheme for injection and acceleration of electrons in wakefield accelerators is suggested based on the co-action of a laser pulse and an electron beam. This synergy leads to stronger wakefield generation and higher energy gain in the bubble regime. The strong deformation of the whole bubble leads to electron self-injection at lower laser powers and lower plasma densities. To predict the practical ranges of electron beam and laser pulse parameters an interpretive model is proposed. The effects of altering the initial electron beam position on self-trapping of plasma electrons are studied. It is observed that an ultra-short (25 fs), high charge (340 pC), 1 GeV electron bunch is produced by injection of a 280 pC electron beam in the decelerating phase of the 75 TW laser driven wakefield.

## Introduction

Acceleration of electrons to relativistic energies in the blowout regime of the laser wakefield accelerators in very short distances is one of the most promising schemes^1–6^. In this technique, the ponderomotive force of the laser pulse forms extremely nonlinear wakefields by expelling the whole background plasma electrons and generating a near-spherical cavitated region that supports strong accelerating and focusing fields^7^. Providing a path to produce ultra-short high energy electron bunches using different methods of injecting electrons in a plasma wakefield^8–11^ and accelerating them to GeV range^12,13^ has drawn wide attention in plasma based accelerator researches recently.

The basic requirements to achieve ultra-short multi-GeV electron bunches are first producing a high gradient accelerating field and then the injecting of electrons into these accelerating structures. As it is known, lower plasma densities enhance the maximum energy of the electrons because at lower densities the phase velocity of the wake is close to the light velocity c, thereby the dephasing length increases^14^. However, it is necessary to use higher laser pulse energy and longer accelerator lengths in this regime and therefore, these conditions make the guiding of the laser pulse more difficult^15^. Furthermore, the high-charge self-trapping of plasma electrons might be challenging in low-density plasmas.

For self-guided propagation of laser pulses, the laser power must be higher than a critical power, *P* ≥ *P*_*c*_^16^, where $P_{c} = 17 \omega_{0}^{2} / \omega_{p}^{2} \left[ {GW} \right]$, *ω*_0_ and *ω*_*p*_ are laser frequency and plasma frequency, respectively. This indicates that as the electron density decreases we need to increase the laser intensity to maintain laser self-guiding because the technology for making the meter-scale plasma channels for external guiding is not developed yet. Although, as described in Refs.^17–19^, the ponderomotive force exerted by a laser pulse causes the electron density to decrease and the electron relativistic mass to increase and, consequently, the plasma refractive index is modified and the leading edge of the laser pulse, locally pump depletes. The rate of etching of the laser pulse energy will be faster for higher intensities. Eventually, the more the laser pulse intensity is, the faster it will lose its energy and then cannot be self-guided in a plasma and excite a stable wake for a long distance. On the other hand, the laser pulse intensity cannot be too large if one needs high efficiency^19^ and thus, using a moderately large intensity, short-pulse laser produces a more stable accelerating structure. However, wakefield strength depends on the input laser power. Therefore, representing new methods for generating stronger wakefield structures and subsequently extending the electron beam energy gain for moderate laser intensities seems an essential subject in the development of plasma-based accelerators.

A mode transition from a laser wakefield accelerator (LWFA) scheme to a plasma wakefield accelerator (PWFA) scheme in a single-stage plasma is already proposed and using these hybrid accelerators results in higher energy gain^20,21^. Injecting electron beams produced or accelerated in a first stage to the second stage that is driven by a different laser pulse is investigated using simulations and experimentally in multi-stage accelerators before^22,23^. Employing these methods leads to considerable improvement in the final energy of the electron beam. This paper propose a new paradigm for electron self-injection and enhancing electron energy gain in plasma accelerators. In this work, the stable guiding of an intense laser pulse which is followed by a short electron bunch is considered. An electron bunch is externally injected into the decelerating field of the plasma bubble and severely modifies the structure of the plasma wakefield. Any low-quality electron beam can be used in this scheme. By injecting an electron beam to the deceleration phase of the laser driven plasma wakefield, the bubble as a whole is strongly affected. The modification of the wake strengthen the laser wakefield accelerating phase (∼ 180 GeV/m) and energy gain (∼ 250 MeV) is considerably enhanced in comparison with using each of the laser wakefield accelerators or the plasma wakefield accelerator individually. In addition, the bubble expansion results in the self-trapping of the plasma electrons.

Electron injection via a plasma density down-ramp^24^, ionization-induced injection in a distinct region^13^ or by channeling laser pulse in a plasma waveguid^25^ and accelerating them to GeV range energy in a low density plasma is studied in plasma accelerators. However, the high-charge self-trapping of plasma electrons at low plasma densities and accelerating them to GeV range for presently available laser systems can be challenging. Therefore, generating quasi-mono-energetic beams of electrons from plasmas are considered as an important concern. We also report a new scheme for plasma electron self-trapping into the plasma wakefield using a hybrid accelerator which happens at lower laser powers and densities than would be possible. The phase slippage (dephasing) between the decelerating electric field of the bubble and the injected electron beam (electron beam velocity is higher than wake velocity in a plasma) and the electron beam density variation in the focusing field region of the laser-driven bubble and the laser pulse field, leads to bubble evolves constantly and electron injection triggers^26–28^. The quality, energy, and length of the self-injected electrons are studied in this paper. The effects of the primary electron beam initial position on self-injection of plasma electrons are investigated. Employing particle-in-cell (PIC) simulations, it is figured out by injection of a 280 pC bunch in the decelerating phase of 75 TW laser wakefield an ultra-short (25 fs), high charge (340 pC), 1 GeV electron bunch is produced.

## Results

### An interpretive model to determine applicable laser and beam parameters for a hybrid accelerator

We first must represent a way to specify appropriate electron bunch parameters in the hybrid scheme which lead to the optimum condition to maximize witness electron energy gain. In fact, the applicability of injecting an external charge in the decelerating phase of the LWFAs is limited due to transition to the beam-dominated regime. In this regime electrons trajectories in bubble sheath and, in consequence, the wakefield strength dominantly determined by the electron beam.

We first start with the quantitative analysis of the bubble which has an electron bunch in its decelerating phase. It is possible to characterize bubble shape by the trajectory of innermost electrons sheath surrounding the near-spherical cavity^29,30^. The differential equation which describes this structure is studied extensively in Ref.^30^. In the ultra-relativistic regime, the shape of the sheath is described by

1 $$r_{b} \frac{{d^{2} r_{b} }}{{d\xi^{2} }} + \left( {\frac{{dr_{b} }}{d\xi }} \right)^{2} + 1 = 4\frac{\lambda \left( \xi \right)}{{r_{b}^{2} }} ,$$

where *r*_*b*_(*ξ*) defines normalized bubble radius, *ξ* is the dimensionless coordinate in a frame moving with light velocity, and *λ*(*ξ*) represents the normalized charge per unit length of the load. By direct integrating of Eq. (1) some aspects of the evolution of the excited plasma wakefield that happens due to loading electron bunch in the decelerating portion of the plasma bubble can be analyzed.

**Figure 1:**
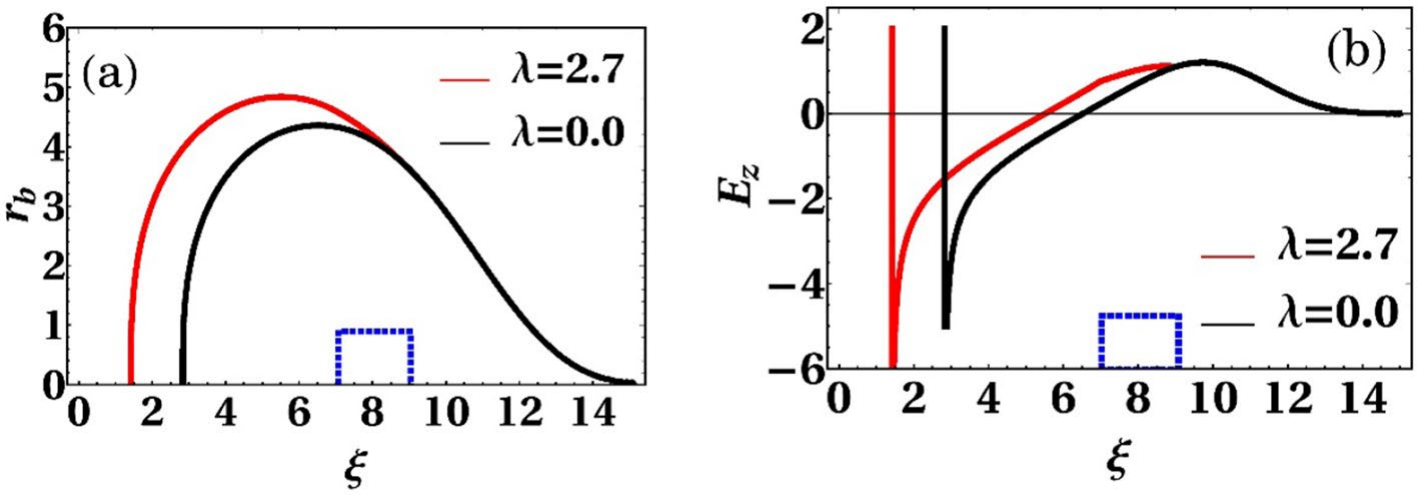
(**a**) Shape of the bubble and (**b**) the corresponding wakefields for maximum blowout radius, r_b_ = 4 (black), and a hybrid laser pulse with maximum blowout radius, r_b_ = 4 and bi-flat-top electron beam with λ = 2.7 (red). The electron beam is located at 7 ≤ ξ ≤ 9 (blue square).

Figure 1 shows the synergistic nature of the hybrid laser and electron beam drivers. It is apparent from Fig. 1a how the bubble structure and its accelerating field, Fig. 1b, are changed after loading a bi-flat-top beam within the front part of the bubble. The presence of electron bunch in the decelerating phase of the wakefield repels the electrons in the sheath around the bubble and makes it bends away. As a result, the bubble absorbs the energy of the beam and consequently, its maximum radius increases and the resulted accelerating field grows up. Witness electrons, traveling at nearly c, phase slip with respect to the laser-driven wake. Therefore, the average field that an electron feels is half of the useful accelerating field peak in a hybrid laser and laser wakefield accelerator^19^. On the other side, witness electrons in the accelerating phase of the PWFAs feels the maximum of the field, because the effect of phase slippage is negligible in this scheme. The useful acceleration field an electron feels in the wake of a low-charged relativistic electron beam is^31^ $E_{{z{ - }\max }} \approx \left( {236\,{\text{MV/ m}} } \right)\left( { N /\left( {4 \times 10^{4} } \right) } \right)\left( { 600\,\upmu {\text{m}} /\sigma_{z} } \right)^{2} \ln \left( { 50\,\upmu {\text{m}} /\sigma_{r} \sqrt {10^{16} \,{\text{cm}}^{ - 3} / n_{0} } } \right)$, where, *n*_0_ is plasma density, *N*, *σ*_*r*_ , and *σ*_*z*_ are the number of electrons, transverse spot size, and electron beam length, respectively. Hence, using this analysis, the electron and laser driver parameters can be determined by ensuring that, the average field which an electron feels in a hybrid accelerator is larger than the maximum PWFAs accelerating field for the same acceleration length.

### Effects of laser pulse-electron beam synergy on the bubble structure and accelerating fields

To study hybrid laser and electron beam accelerators and checking the consequence of injecting an electron bunch into the decelerating phase of LWFAs, we perform a series of 2D simulations using particle-in-cell (PIC) code OSIRIS^32^ (see Methods). The studied case consisted of co-propagating of a 75 TW laser pulse and an electron beam in a plasma with electron density, n_0_ = 3.4 × 10^18^ cm^−3^. The electron beam is initially located 3 µm behind the peak of the laser.

**Figure 2:**
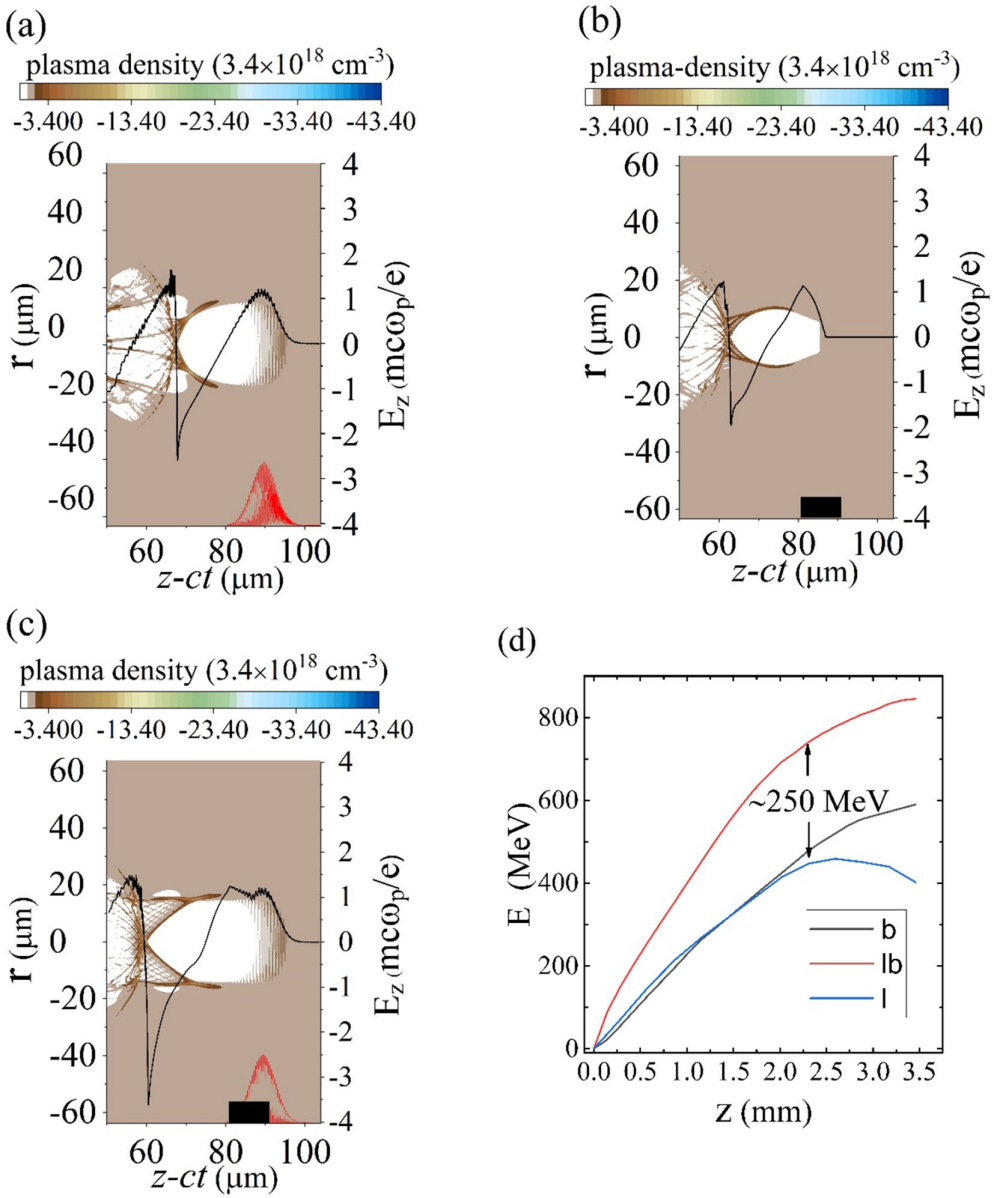
Background density snapshots and corresponding wakefield after 0.1 mm propagation distance for (**a**) LWFAs, (**b**) PWFAs and (**c**) hybrid laser and electron beam accelerators. Red and black lines show laser pulse and electron beam drivers location respectively. (**d**) Energy gain from the wake of the laser driver (blue), electron driver (black), and hybrid driver (red) for 3.5 mm propagation distance.

To compare the synergistic nature of the laser pulse-electron beam wakefield accelerators with LWFAs and PWFAs, we displayed snapshots of the background plasma density and corresponding wake electric field at propagation distance of 0.1 mm for a LWFA (Fig. 2a), a PWFA (Fig. 2b), and a hybrid laser and electron beam driver (Fig. 2c). It is obvious, how the initial injection in the decelerating phase makes the whole bubble becomes larger. As a result of whole bubble expansion, stronger charge separation occurs, and accordingly, the corresponding accelerating field increases substantially. The co-action of laser and electron beam produces a peak gradient of 530 GeV/m which is about 180 GeV/m larger than each laser or beam driver accelerating field. The transformer ratio (R) which is defined as the maximum accelerating field to the maximum decelerating field increases to R = 3.2 for a hybrid accelerator which is enhanced by a factor of 1.5 and 1.8 compare with LWFAs and PWFAs, respectively. Energy gain (E) of test electrons with initial energy of 700 MeV in these three regimes are compared in Fig. 2d for 3.5 mm propagation distance. As it is shown in Fig. 2d, electrons gain more energy, approximately 250 MeV, in a hybrid accelerator. Practically, acceleration length is limited to 2.3 mm because the electrons in a beam driver lose their energy by the wakefield and start slipping backward by passing the acceleration length. On the other hand, the pump depletion effect causes the laser pulse diffraction, so it cannot be self-guided anymore, Fig. 2d.

### Electron self-trapping mechanism and the energy and quality of injected electrons

The technique of using the hybrid laser pulse and beam driver introduces a new scheme for the plasma electron self-injection. In the rest of this paper, we will investigate this new self-injection scenario. Injecting an electron bunch in the deceleration phase of the bubble makes the bubble radially and longitudinally expands. This extension reduces the gamma-factor of the bubble. In addition, the wakefield gets stronger as a result of the beam and laser synergy, so the trapping of the plasma background electrons at lower plasma densities and lower laser powers becomes attainable. By specifying the appropriate range of the parameters and the initial injection position for the electron bunch driver, producing a high amount of charge and low energy spread electron beam is possible.

**Figure 3:**
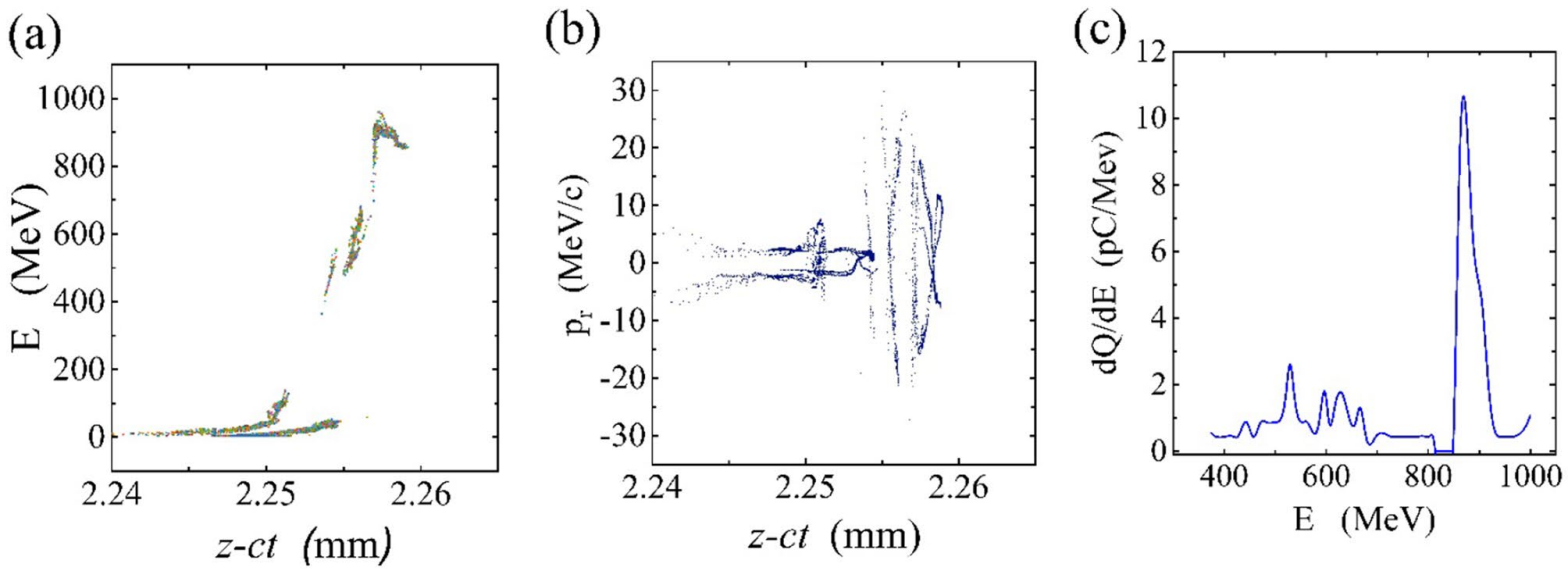
The phase space of self-trapped electrons for a hybrid accelerator after 2.3 mm propagation. Energy gain from the wake (**a**), Transverse momentum vs. propagation distance (**b**), and electron energy spectrum (**c**).

To illustrate the quality, charge, and energy of self-injected electrons, we plotted phase space and energy spectrum of them in Fig. 3 at propagation distance 2.3 mm. Figure 3 represents two kinds of self-trapped electrons, a peak with 340 pC of charge and an electron tail that carries 60 pC of charge. Thus, the electron peak forms the principal part of the self-injected beam. The self-injection process ends when the electron bunch reaches the front part of the laser pulse. The self-trapped electrons are produced by transverse injection mechanism^33^. They are mostly originated from electron sheath around the bubble. As Fig. 3b confirms the peak electrons have larger transverse momentum while electrons in the tail experience smaller transverse motion. The main peak of accelerated electrons reaches 900 MeV energy with rms spread of 6.6% and a duration of 25 fs. Therefore, combining an electron bunch of 270 pC with a laser pulse generates an electron beam with larger amounts of charge.

**Figure 4:**
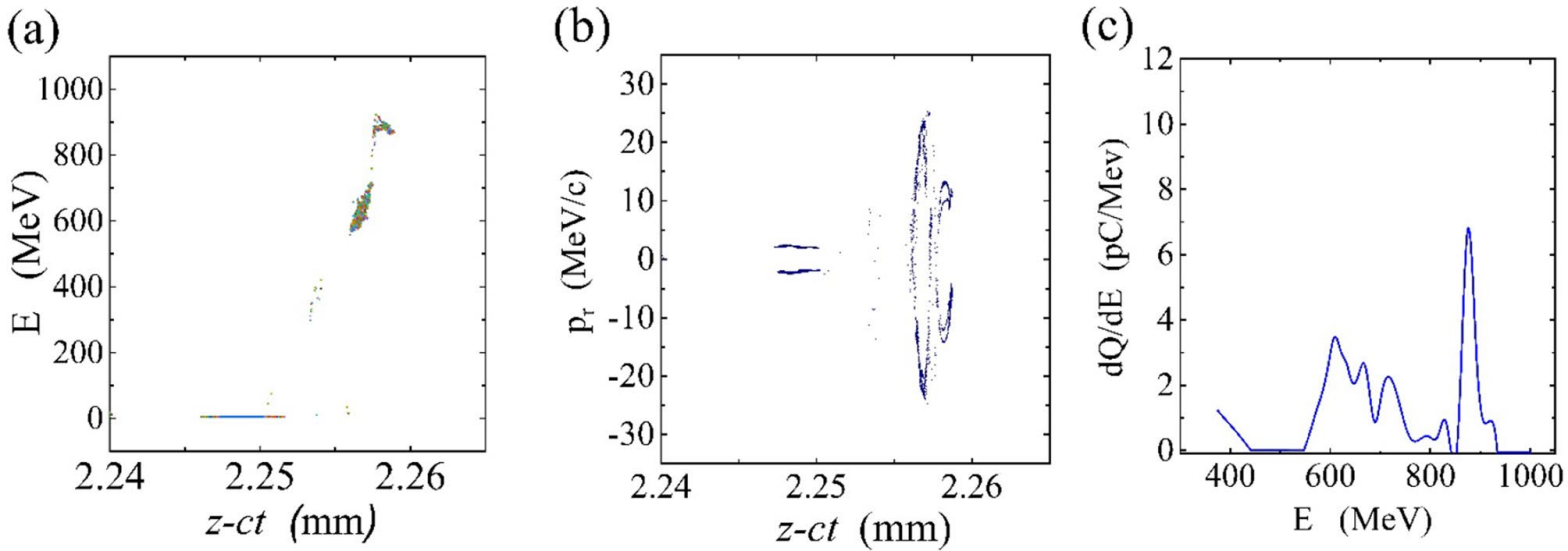
The phase space of self-trapped electrons for a hybrid accelerator considering a non-evolving electron bunch after 2.3 mm propagation. Energy gain from the wake (**a**), Transverse momentum vs. propagation distance (**b**), and electron energy spectrum (**c**).

A better physical understanding of the causes of the bubble expansion and the electron self-injection is possible by considering that two phenomena can affect the bubble structure. First, the primary electron bunch slips forward with respect to the laser pulse and second, the electron bunch density variation because it is in the strong focusing field region and interacts with the laser pulse field. To explain the role of each part in a hybrid accelerator, we considered the injection of a non-evolving electron beam in the deceleration phase of a laser-driven wakefield; thus, the effect of the density variation of the electron bunch is negligible (Fig. 4). Studying of non-evolving electron beam represents about 240 pC of charges are self-trapped in the peak and the tail of the self-injected bunch. It is 100 pC less than the peak of the condition in which the beam density variation in focusing and laser fields is considered. Figure 4 represents the phase slippage between the electron beam and the laser pulse plays a more important role in the self-injection mechanism. In fact, the electron beam feels a strong focusing field but simultaneously it is interacting with laser pulse field that makes the electrons gain large transverse momentum.

### The role of the initial electron beam position

The initial beam position makes a major contribution in the total charge of the self-injected electrons in this scheme. Choosing the appropriate electron beam location is possible by considering two points. First, as Fig. 1a shows the slope of the electron sheath around the bubble is much larger and the bubble radius (r_b_) is smaller behind the laser pulse. If we define the position of the electron beam in this region, the interaction between electron bunch and the electrons in the sheath will get stronger during propagation. The reason is that, the bubble radius (r_b_) gets smaller and smaller during the phase slippage process. Second, if we place the electron beam initial position very close to the head of the laser pulse, the driver beam catches up to the head of the laser pulse. This will result in removing electron beam from the bubble quickly, therefore, it cannot change the bubble structure anymore. By considering the points are described the electron beam and the laser pulse synchronization is possible.

**Figure 5:**
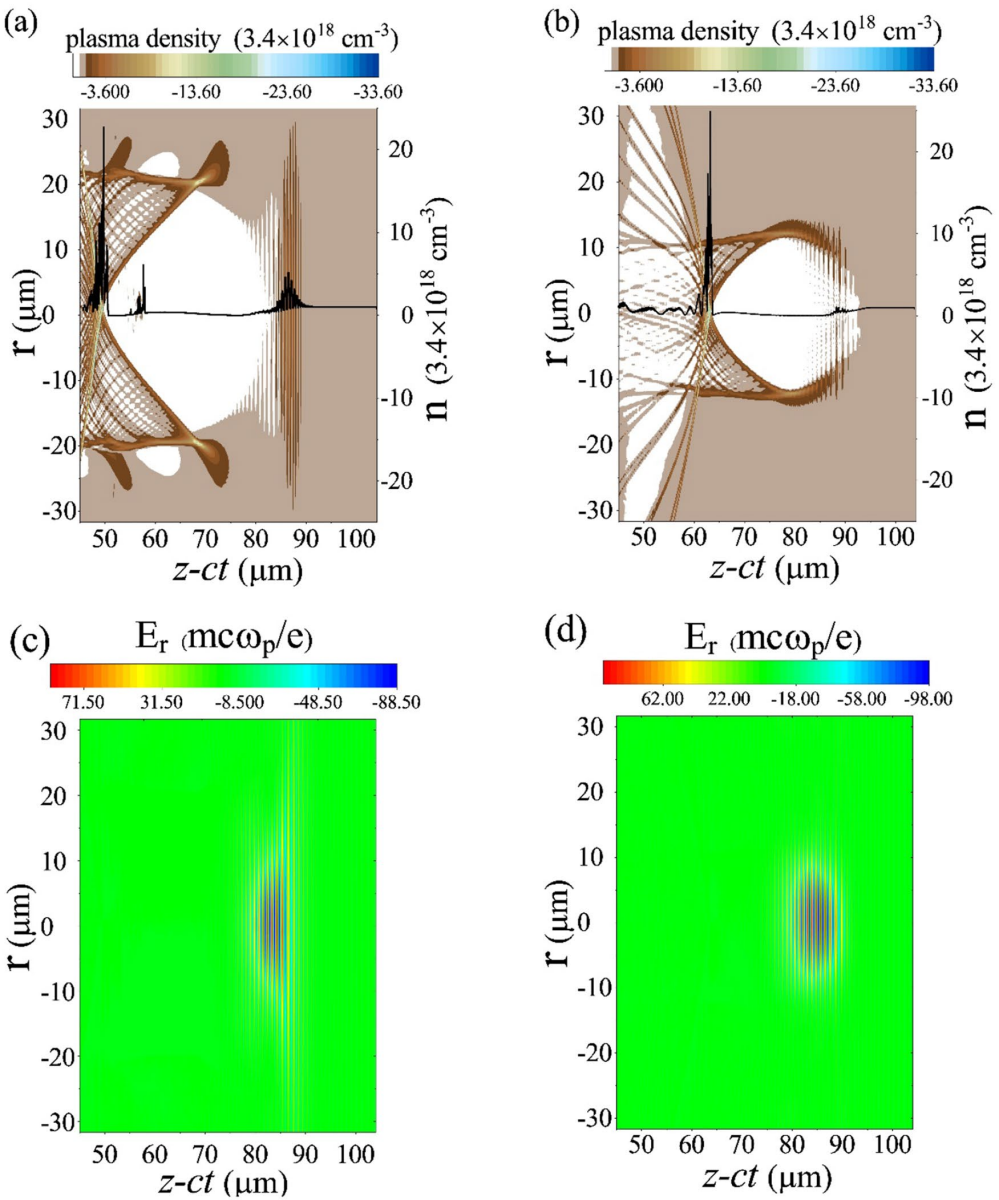
Effects of initial electron beam position on electron self-injection and laser self-guiding at 2.3 mm propagation distance. (**a**) Plasma electron density snapshot and density distribution on the propagation axis (black line). The electron beam is initially located 3 μm behind the laser pulse peak. (**b**) Plasma electron density snapshot and density distribution on the propagation axis (black line). The electron beam is initially located 5.8 μm ahead of the laser pulse peak. (**c**) Transverse laser pulse profile. The electron beam is initially located 3 μm behind the laser pulse peak. (**d**) Transverse laser pulse profile. The electron beam is initially located 5.8 μm ahead of the laser pulse peak.

Figure 5a shows, placing the electron beam behind the peak of the laser pulse gives rise to the self-trapping of the electrons. This is because the presence of an electron beam in the decelerating part of the wakefield makes an evolving bubble as discussed. However, if the electron beam is located in the front part of the laser pulse peak, it will contribute more to repelling plasma electrons outward which leads to form the wider plasma ion channel behind. Then it cannot play a significant role in deforming the bubble structure, Fig. 5b. Figure 5d confirms when the electron beam is located in the front part of the pulse, the laser stayed more focused and as a result can be self-guided for longer propagation distances^34^. Although, the laser pulse is almost diffracted in the same propagation distance when the electron beam is behind the peak of the laser pulse (Fig. 5c).

## Discussion

In conclusion, a new type of wakefield accelerator using laser pulse and electron beam synergy is presented. An interpretive model is introduced to determine applicable laser and beam parameters. In this scheme, the injection of 280 pC electron beam charge in decelerating part of the laser-driven wakefield causes the bubble to expand, which strengthens the accelerating field and gives rise to self-injection at lower plasma densities and lower laser powers than is possible without this method. The self-injected electron beam has 340 pC of charge and reaches 900 MeV energy with rms spread of 6.6%. The witness beam gained 250 MeV more energy in a hybrid accelerator compare with LWFAs and PWFAs. The self-trapped electrons are very short and they accelerate to the near-GeV range with high charges. Studying the initial electron beam position demonstrated that electron self-trapping occurs if the beam is located behind the peak of the laser pulse.

## Methods

Particle-in-cell simulations. Simulation results shown in the paper were performed with the particle-in-cell code OSIRIS^32^. We consider the bubble regime of electron acceleration. A 30 fs (FWHM), 0.8 µm, 75 TW laser beam is focused to a Gaussian diffraction-limited spot size of w_0_ = 11.52 µm which corresponds to normalized vector potential of a_0_ = 4 at the entrance of plasma density n_0_ = 3.4 × 10^18^ cm^−3^. The parameters of the electron beam are: k_p_σ_z_ = 2.0, k_p_σ_r_ = 1.5 and n_0b_/n_0_ = 1.2, where n_0b_ is electron beam peak density and k_p_ is plasma wave number. The electron total charge is estimated at about 280 pC and it is behind the peak of the pulse. Initial electrons energy is 700 MeV. The simulation window moves at light velocity (c) along the laser propagation direction and have dimensions of 104 µm × 126.7 µm. The number of grid points is 6500 × 350. Four macro-particles per cell is considered for the plasma and 10 macro–particles for the beam. Numerical solution of the electrons trajectories equation. As shown in Fig. 1, the equation of electrons trajectories for a hybrid accelerator (Eq. 1) is solved by numerical direct integrating for a laser pulse with normalized vector potential a_0_ = 4 and an electron beam λ = 2.7.

## Data Availability

The data that supports the results of this study is available from the corresponding author upon reasonable request.
